# PIAS1 alleviates diabetic peripheral neuropathy through SUMOlation of PPAR-γ and miR-124-induced downregulation of EZH2/STAT3

**DOI:** 10.1038/s41420-021-00765-w

**Published:** 2021-12-02

**Authors:** Zixin Hou, Ji Chen, Huan Yang, Xiaoling Hu, Fengrui Yang

**Affiliations:** 1 Department of Anesthesiology, The First Affiliated Hospital of University of South China, Hengyang, 421001 P.R. China; 2 Department of Endocrinology, The First Affiliated Hospital of University of South China, Hengyang, 421001 P.R. China; 3 Department of Anesthesiology, Affiliated Huaihua Hospital, University of South China, Huaihua, 418000 P.R. China

**Keywords:** Diseases, Cancer

## Abstract

Diabetic peripheral neuropathy (DPN) is a frequently occurring chronic complication of diabetes. In this study, we aim to explore the regulatory mechanism of protein inhibitor of activated STAT1 (PIAS1) in DPN in terms of autophagy and apoptosis of Schwann cells. The SUMOlation of PPAR-γ by PIAS1 was examined, and ChIP was performed to verify the binding of PPAR-γ to miR-124 promoter region. Dual-luciferase gene reporter assay was used to validate the binding affinity between miR-124 and EZH2/STAT3. Following loss‐ and gain‐of-function experiments, in vitro assays in high glucose-treated Schwann cells (SC4) and in vivo assays in db/db and ob/ob mice were performed to detect the effects of PIAS1 on autophagy and apoptosis of Schwann cells as well as symptoms of DPN by regulating the PPAR-γ-miR-124-EZH2/STAT3. The expression of PIAS1, PPAR-γ, and miR-124 was downregulated in the sciatic nerve tissue of diabetic mice. PIAS1 enhanced the expression of PPAR-γ through direct binding and SUMOlation of PPAR-γ. PPAR-γ enhanced the expression of miR-124 by enhancing the promoter activity of miR-124. Furthermore, miR-124 targeted and inversely modulated EZH2 and STAT3, promoting the autophagy of Schwann cells and inhibiting their apoptosis. In vivo experiments further substantiated that PIAS1 could promote the autophagy and inhibit the apoptosis of Schwann cells through the PPAR-γ-miR-124-EZH2/STAT3 axis. In conclusion, PIAS1 promoted SUMOlation of PPAR-γ to stabilize PPAR-γ expression, which upregulated miR-124 to inactivate EZH2/STAT3, thereby inhibiting apoptosis and promoting autophagy of Schwann cells to suppress the development of DPN.

## Introduction

Diabetic peripheral neuropathy (DPN) is a common complication of diabetes mellitus [1]. DPN may lead to nerve injury and muscle strength decrease in patients [2] and can result in autonomic dysfunction [3]. The pathogenesis of DPN is a complicated process involving multiple factors [4]; and, notably, injury to Schwann cells has been highlighted in the progression of DPN [5–7]. A previous review has indicated that Schwann cell apoptosis is induced by high glucose and is involved in the pathogenesis of DPN [8]. Cheng et al. also stated that hyperglycemia induces oxidative stress and inflammatory responses that damage nerve tissue, and that Schwann cell dysfunction could further promote the progression of DPN [6]. Moreover, Schwann cell apoptosis induced by endoplasmic reticulum stress has been suggested to be one of the main pathogeneses of DPN [9]. The relationship between the activities of Schwann cells and DPN is not well-defined. Knowledge of the events of Schwann cell apoptosis and autophagy may allow for earlier diagnoses and novel therapies.

Protein inhibitor of activated STAT1 (PIAS1) is identified as a small ubiquitin-like modifier (SUMO) E3 ligase that regulates a variety of cellular processes including cell proliferation, DNA damage, as well as inflammatory reaction [10]. Notably, it has been revealed that PIAS1 exerted important functions in controlling insulin sensitivity and thus might be potential therapeutic target for treatment of type 2 diabetes mellitus [11]. It has been unveiled that PIAS1 can induce the SUMO modification of peroxisome proliferator-activated receptor γ (PPAR-γ) to prevent myocardial ischemia-reperfusion injury [12]. PPAR-γ belongs to the nuclear hormone receptor transcription factor family [13]. Intriguingly, PPAR-γ could induce differentiation of Schwann cells, thereby regulating injury and regeneration of peripheral nerves [14].

Of note, activated PPAR-γ was found to upregulate miR-124 in patients with sepsis, which contributed to the suppression of production of pro-inflammatory cytokines [15]. Inhibition of miR-124 could reverse the alleviation of neuropathic pain in diabetic rats [16]. As previously reported, miR-124 could target the EZH2-STAT3 axis in cholangiocarcinoma cells [17]. Enhancer of zeste homolog 2 (EZH2) is considered to be a methyltransferase with the ability of di- and tri-methylating lysine-27 of histone H3 [18]. Notably, EZH2 was found to aid in the promotion of fibrosis of diabetic nephropathy [19]. Signal transducer and activator of transcription 3 (STAT3) is regarded as a transcription factor participating in the transmission of extracellular signal to the nucleus [20]. The phosphorylation of STAT3 could regulate the impaired autophagy of Schwann cells due to high glucose in DPN [21]. Considering all the above findings, we formulate a hypothesis in the current study that PIAS1 is likely to regulate the development of DPN, with the involvement of the PPAR-γ-miR-124-EZH2/STAT3 axis.

## Results

### PIAS1 was downregulated in the nerve tissues of diabetic mice, and overexpression of PIAS1 enhanced the autophagy of Schwann cells

**Fig. 1:**
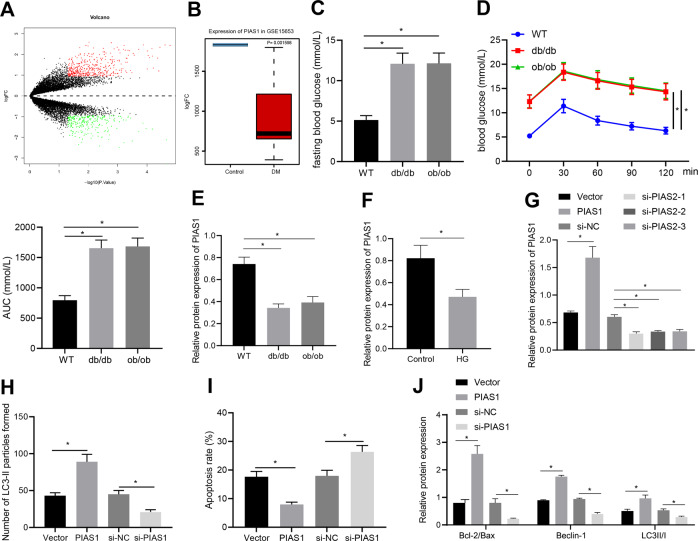
PIAS1 is downregulated in the nerve tissues of diabetic mice, and overexpression of PIAS1 promotes the autophagy of Schwann cells. **A** The heatmap for gene analysis of gene expression based on the GSE15653 dataset. The red dots indicate significantly upregulated genes, the green dots indicated significantly downregulated genes, and the black dots indicate genes with no significant difference. **B** The expression box diagram of PIAS1 in the GSE15653 dataset, and the significance of the difference is shown in the upper right corner. **C**, **D** Six male C57BL/6 WT mice, ob/ob mice, and db/db mice aged 8 weeks old were selected to detect the fasting blood glucose (**C**) and were subjected to OGTT test (**D**). **E** PIAS1 protein expression as determined by western blot assay (*n* = 6). **F** SC4 cells were treated with high glucose and western blot assay was performed to determine PIAS1 protein expression. **G** After overexpression or knockdown of PIAS1 in SC4 cells, western blot assay was performed to determine the overexpression and knockdown efficiency, and the one with the highest knockdown efficiency was selected for follow-up experiments. **H** LC3-II immunofluorescence staining. **I** The apoptosis after overexpression or knockdown of PIAS1 as examined by flow cytometry. **J** The expression of apoptosis and autophagy-related proteins as determined by western blot assay. *p < 0.05. Data between two groups were compared by independent sample *t* test, and those among multiples by one-way ANOVA, followed by Tukey’s post hoc tests. The experiment was conducted in triplicates.

Through the differential analysis of the diabetes-related dataset GSE15653 downloaded from the GEO database (https://www.ncbi.nlm.nih.gov/gds), we obtained 426 significantly upregulated genes and 262 significantly downregulated genes (Fig. 1A). We found that PIAS1 was notably downregulated in diabetes mellitus (Fig. 1B). We speculated that PIAS1 may be a key gene affecting diabetic neuropathy. In order to explore whether PIAS1 plays a role in DPN, we first selected db/db mice and ob/ob mice to establish spontaneous diabetes mellitus model, and used WT C57BL/6 mice as NCs. Fasting blood glucose was measured and oral glucose tolerance test (OGTT) was carried out to confirm the establishment of diabetic models in db/db mice and ob/ob mice (Fig. 1C, D). The expression of PIAS1 in sciatic nerve tissues of the mice was determined. The results showed that compared with control mice, db/db, and ob/ob diabetic mice exhibited markedly decreased protein expression of PIAS1 in the sciatic nerve tissues (Fig. 1E). Moreover, the protein expression of PIAS1 was also decreased in SC4 cells treated with high glucose (Fig. 1F). To verify whether PIAS1 affects autophagy and apoptosis of peripheral nerves, PIAS1 was overexpressed or knocked down in SC4 cells treated with high glucose (Fig. 1G). Based on the results from immunofluorescence staining, flow cytometry, and western blot assay, overexpression of PIAS1 increased cell autophagy activity and inhibited apoptosis, accompanied by increased expression of B-cell leukemia/lymphoma 2 (Bcl-2)/Bax, Beclin-1, and light chain 3(LC3)II/I, while these effects were reversed by knockdown of PIAS1 (Fig. 1H–J). In summary, the above results revealed the downregulation of PIAS1 in the nerve tissues of diabetic mice, and the enhanced autophagy of SC4 cells by overexpression of PIAS1.

### PIAS1 enhanced miR-124 expression through SUMOlation of PPAR-γ

**Fig. 2:**
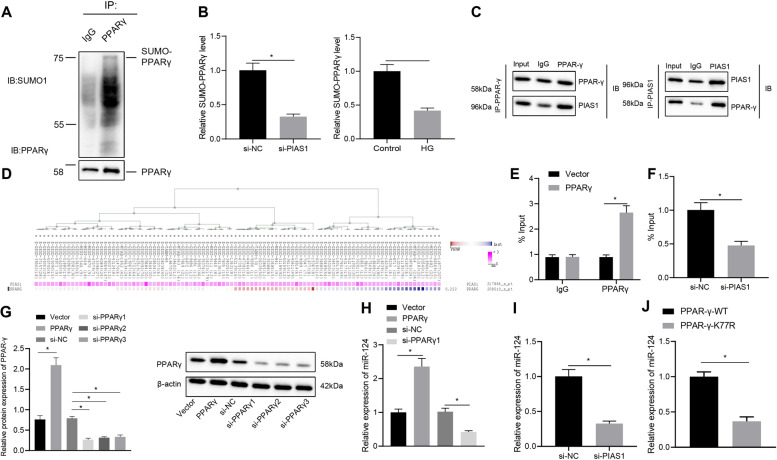
PIAS1 promotes miR-124 expression through SUMOlation of PPAR-γ. **A** The SUMOlation of PPAR-γ as detected by Co-IP. **B** The SUMOlation of PPAR-γ after PIAS1 was knocked down or treated with high glucose in SC4 cells as detected by Co-IP. **C** The interaction between PIAS1 and PPAR-γ as detected by Co-IP. **D** The significant co-expression of PIAS1 and PPAR-γ as detected by MEM analysis. **E** The binding of PPAR-γ with miR-124 promoter as detected by ChIP. **F** The binding of PPAR-γ with miR-124 promoter after PIAS1 knockdown as detected by ChIP. **G** The expression of PPAR-γ in cells after PPAR-γ knockdown or overexpression as determined by western blot assay. The one with the best knockdown efficiency was selected for subsequent experiments. **H** The expression of PPAR-γ in cells after PPAR-γ knockdown or overexpression as determined by RT-qPCR. **I** The expression of miR-124 in cells after PPAR-γ knockdown or overexpression as determined by RT-qPCR. **J** qRT-PCR measurement of miR-124 expression in cells treated with PPAR-γ or mutant PPAR-γ-K77R vectors. *p < 0.05. Data between two groups were compared by independent sample *t* test, and those among multiples by one-way ANOVA, followed by Tukey’s post hoc tests. The experiment was conducted in triplicates.

We investigated whether PIAS1 could regulate the SUMOylation of PPAR-γ in DPN. First, SUMOlation of PPAR-γ was successfully detected by Co-IP using PPAR-γ antibody (Fig. 2A). Knocking down PIAS1 or high glucose treatment was then unraveled to repress the SUMO binding on PPAR-γ in SC4 cells (Fig. 2B). Further to verify the interaction between PIAS1 and PPAR-γ, we transduced PIAS1 and PPAR-γ plasmids into SC4 cells, respectively, and Co-IP results revealed that PIAS1 interacted with PPAR-γ (Fig. 2C). Through MEM (https://biit.cs.ut.ee/mem/index.cgi), we found that MEM, PIAS1, and PPAR-γ were significantly co-expressed (Fig. 2D). We thus speculated that PIAS1 may affect diabetic peripheral nerves through PPAR-γ mediated miR-124 expression in relation to PPAR-γ SUMOlation. Moreover, ChIP results showed that PPAR-γ bound to miR-124 promoter regions (Fig. 2E), and that the binding was weakened in the presence of PIAS1 knockdown (Fig. 2F). Furthermore, RT-qPCR displayed that overexpression of PPAR-γ enhanced the expression of miR-124, and knockdown of PPAR-γ inhibited it (Fig. 2G, H). Similarly, knockdown of PIAS1 also reduced the expression of miR-124 (Fig. 2I). Moreover, vectors carrying the wild type (WT) PPAR-γ and mutant PPAR-γ-K77R (mutated at the SUMOylation site) were transfected into cells, respectively, and qRT-PCR results showed that the mutant PPAR-γ-K77R vector led to reduced miR-124 expression (Fig. 2J). Taken together, these results suggested that PIAS1 stabilized PPAR-γ by contributing to the SUMOlation of PPAR-γ, thereby promoting the expression of miR-124.

### PIAS1 enhanced autophagy of Schwann cells via miR-124

**Fig. 3:**
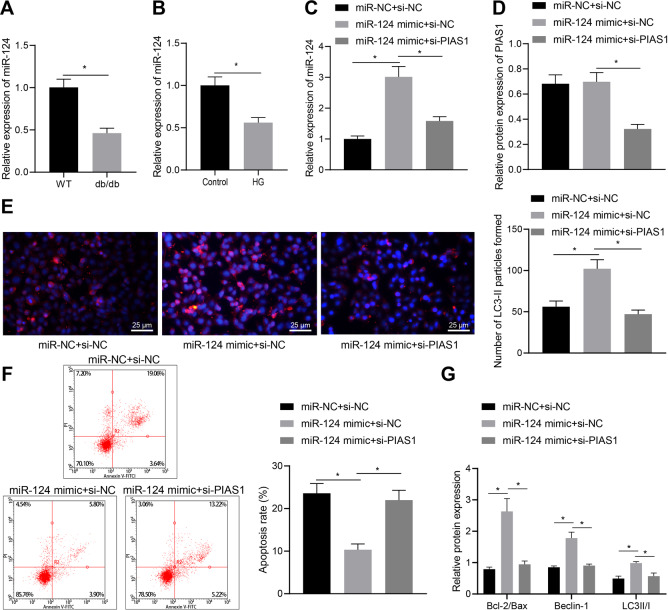
PIAS1 promotes autophagy of Schwann cells via miR-124. **A** The expression of miR-124 in sciatic nerve tissues of C57BL/6 mice and db/db mice as determined by RT-qPCR. **B**, **C** SC4 cells were treated with high glucose, and the expression of miR-124 was detected by RT-qPCR. **D** SC4 cells were treated with high glucose, and the expression of PIAS1 was detected by western blot assay. **E** The number of LC3-II granules as measured by immunofluorescence staining. **F** The proportion of apoptosis as detected by flow cytometry. **G** The expression of apoptosis and autophagy-related proteins as determined by western blot assay. *p < 0.05. Data between two groups were compared by independent sample *t* test, and those among multiples by one-way ANOVA, followed by Tukey’s post hoc tests. The experiment was conducted in triplicates.

Subsequently, we further studied the effect of miR-124 on DPN. It was found that the expression of miR-124 in animal and cell models was decreased (Fig. 3A, B), indicating the low expression of miR-124 in DPN. To verify that PIAS1 affects apoptosis and autophagy through miR-124, we transfected miR-124 mimic into SC4 cells treated with high glucose while knocking down PIAS1 expression. The results displayed that the expression of miR-124 was upregulated in response to miR-124 mimic, and the expression of PIAS1 had no significant change. Further, qRT-PCR and western blot results indicated that the combination of miR-124 overexpression and PIAS1 interference led to decreased miR-124 and PIAS1 expression as compared with miR-124 overexpression alone (Fig. 3C, D). The results of immunofluorescence staining, flow cytometry, and western blot assay showed that miR-124 overexpression increased cell autophagy activity and inhibited apoptosis, as reflected by elevated expression of Bcl-2/Bax, Beclin-1, and LC3II/I, and these effects were abrogated when it was combined with PIAS1 knockdown (Fig. 3E–G). Collectively, these results suggest that PIAS1 can inhibit cell apoptosis and promote autophagy of Schwann cells through miR-124.

### miR-124 enhanced the autophagy of Schwann cells by targeting EZH2 and STAT

**Fig. 4:**
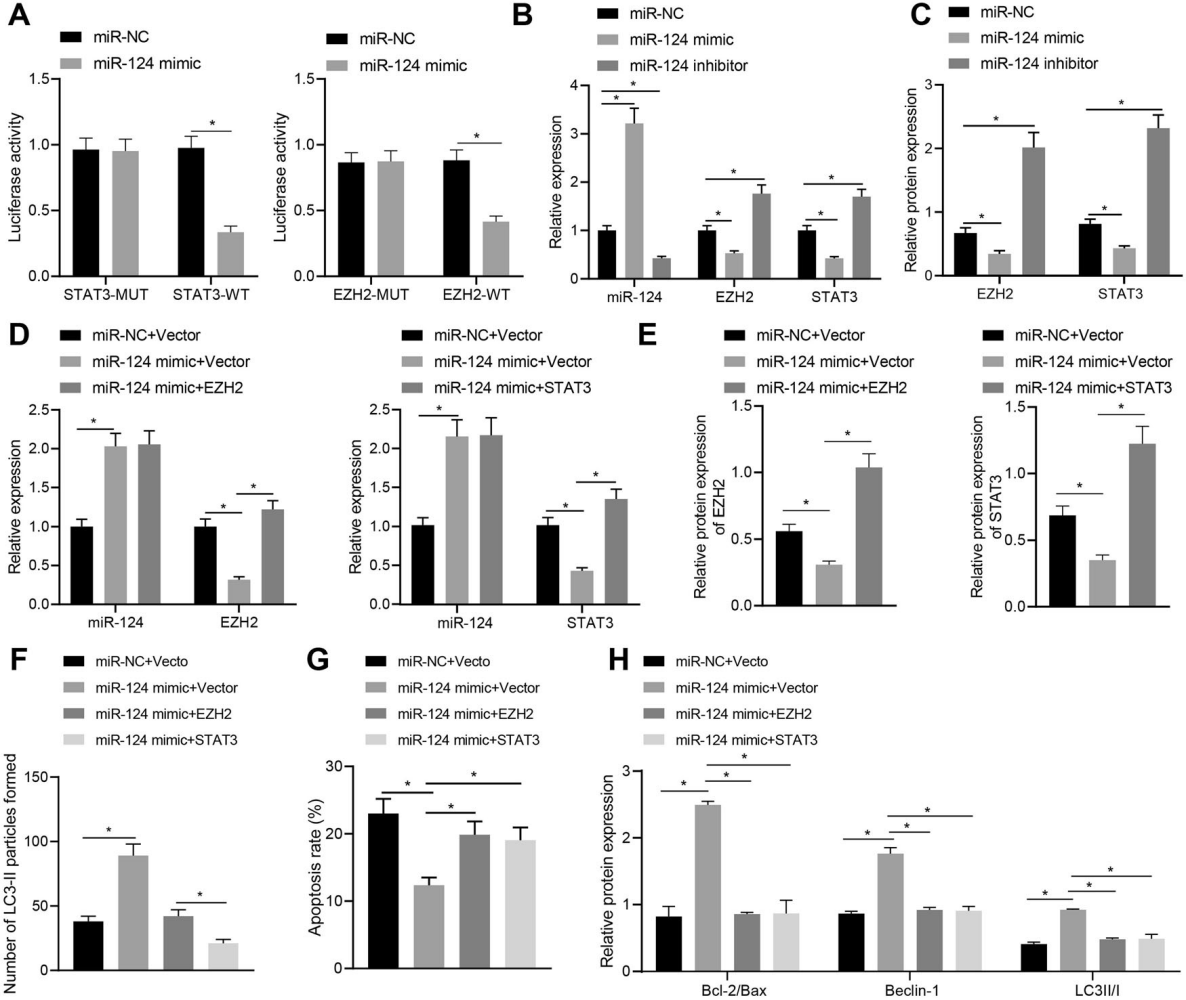
miR-124 promotes the autophagy of Schwann cells by targeting EZH2 and STAT. **A** The binding of miR-124 with EZH2 and STAT3 as verified by dual-luciferase gene reporter assay. **B** The mRNA expression of miR-124, EZH2, and STAT3 in the presence of miR-124 overexpression/knockdown, as determined by RT-qPCR. **C** The protein expression of EZH2 and STAT3 in the presence of miR-124 overexpression/knockdown, as determined by western blot assay. **D** The mRNA expression of miR-124, EZH2, and STAT3 in the presence of miR-124 overexpression alone or in combination with EZH2/STAT3 upregulation, as determined by RT-qPCR. **E** The protein expression of EZH2 and STAT3 in the presence of miR-124 overexpression alone or in combination with EZH2/STAT3 upregulation, as determined by western blot assay. **F** The number of LC3-II granules in cells as detected by immunofluorescence staining. **G** The proportion of apoptosis as detected by flow cytometry. **H** The expression of apoptosis and autophagy-related proteins as detected by western blot assay in the presence of miR-124 overexpression alone or in combination with EZH2/STAT3 upregulation. *p < 0.05. Data between two groups were compared by independent sample *t* test, and those among multiples by one-way ANOVA, followed by Tukey’s post hoc tests. The experiment was conducted in triplicates.

In order to further study the downstream regulation mechanism of miR-124, the RAID 2.0 (http://www.rna-society.org/raid2/index.html) database was used to predict the downstream genes of miR-124. We found that the top three genes were IL6R (score = 0.9999), EZH2 (score = 0.9994), STAT3 (score = 0.9992). The binding of miR-124 to EZH2 and STAT3 was verified by dual-luciferase gene reporter assay in HEK293T cells (Fig. 4A). After transfection of miR-124 mimic or inhibitor into SC4 cells, RT-qPCR and western blot assay results revealed that overexpression of miR-124 could notably inhibit the targeted binding to EZH2 and STAT3, and inhibit the expression of both; while silencing miR-124 led to upregulated expression of EZH2 and STAT3 (Fig. 4B, C). In order to verify whether miR-124 regulates cell function through EZH2 and STAT3, we set up loss‐ and gain‐of‐function experiments in the cell model treated with high glucose. According to RT-qPCR and western blot assay results, miR-124 mimic treatment contributed to an increase in miR-124 expression and a decrease in EZH2 and STAT3 expression. Relative to miR-124 overexpression alone, its combination with EZH2 or STAT3 overexpression resulted in increased expression of EZH2 and STAT3 (Fig. 4D, E). The results of immunofluorescence staining, flow cytometry, and western blot assay revealed that miR-124 mimic treatment increased cell autophagy and inhibited cell apoptosis, accompanied by increased Bcl-2/Bax, Beclin-1, LC3II/I expression, whereas these effects could be negated when it was combined with EZH2/STAT3 overexpression (Fig. 4F–H). Taken together, these results suggest that miR-124 can inhibit the expression of EZH2 and STAT3, thereby inhibiting apoptosis induced by high glucose and promoting autophagy of Schwann cells.

### PIAS1 enhanced autophagy of Schwann cells by regulating miR-124-EZH2/STAT3 axis

**Fig. 5:**
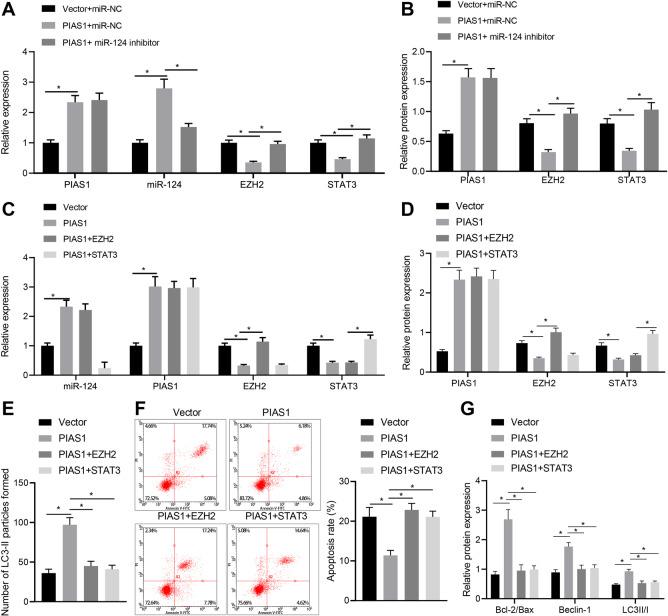
PIAS1 promotes autophagy of Schwann cells by regulating miR-124-EZH2/STAT3 axis. **A** The mRNA expression of miR-124, PIAS1, EZH2, and STAT3 as determined by RT-qPCR. **B** The protein expression of PIAS1, EZH2, and STAT3 in Schwann cells treated with oe-PIAS1 alone or in combination with miR-124 mimic, as determined by western blot assay. **C** The mRNA expression of miR-124, PIAS1, EZH2, and STAT3 in response to PIAS1 overexpression alone or in combination with EZH2/STAT3 upregulation, as determined by RT-qPCR. **D** The protein expression of PIAS1, EZH2, and STAT3 in response to PIAS1 overexpression alone or in combination with EZH2/STAT3 upregulation, as determined by western blot assay. **E** The number of LC3-II particles in cells with different treatment as detected by immunofluorescence staining. **F** The proportion of apoptosis of cells with different treatment as detected by flow cytometry. **G** The expression of apoptosis and autophagy-related proteins in response to PIAS1 overexpression alone or in combination with EZH2/STAT3 upregulation, as determined by western blot assay. *p < 0.05. Data between two groups were compared by independent sample *t* test, and those among multiples by one-way ANOVA, followed by Tukey’s post hoc tests. The experiment was conducted in triplicates.

Based on the above findings, we set out to verify the hypothesis that PIAS1 could regulate the expression of EZH2/STAT3 via PPAR-γ/miR-124. As revealed by RT-qPCR and western blot assay, the expression of PIAS1 and miR-124 was significantly increased while that of EZH2 and STAT3 was decreased in the presence of PIAS1 overexpression. Compared with PIAS1 overexpression alone, its combination with miR-124 inhibition led to no significant change in regard to PIAS1 expression, but decreased expression of miR-124 and increased expression of EZH2 and STAT3. These results suggest that PIAS1 inhibits EZH2/STAT3 expression through miR-124 (Fig. 5A, B). Next, we explored whether PIAS1 affects autophagy of SC4 cells through miR-124-EZH2/STAT3 axis. The results of RT-qPCR and western blot assay revealed that PIAS1 and miR-124 expression was significantly increased while EZH2 and STAT3 expression was markedly decreased in the presence of PIAS1 overexpression. Compared with PIAS1 overexpression, overexpression of PIAS1 and EZH2 increased EZH2 expression, and overexpression of PIAS1 and STAT3 elevated STAT3 expression (Fig. 5C, D). As shown in immunofluorescence staining, flow cytometry, and western blot assay, PIAS1 overexpression increased cell autophagy and inhibited apoptosis induced by high glucose, while elevating Bcl-2/Bax, Beclin-1, and LC3II/I expression; these effects were reversed by overexpression of PIAS1 and EZH2 or overexpression of PIAS1 and STAT3 (Fig. 5E–G). Overall, PIAS1 can inhibit the expression of EZH2 and STAT3 by promoting miR-124, thus inhibiting apoptosis and promoting autophagy of Schwann cells.

### PIAS1 alleviated diabetic neuropathy in vivo

**Fig. 6:**
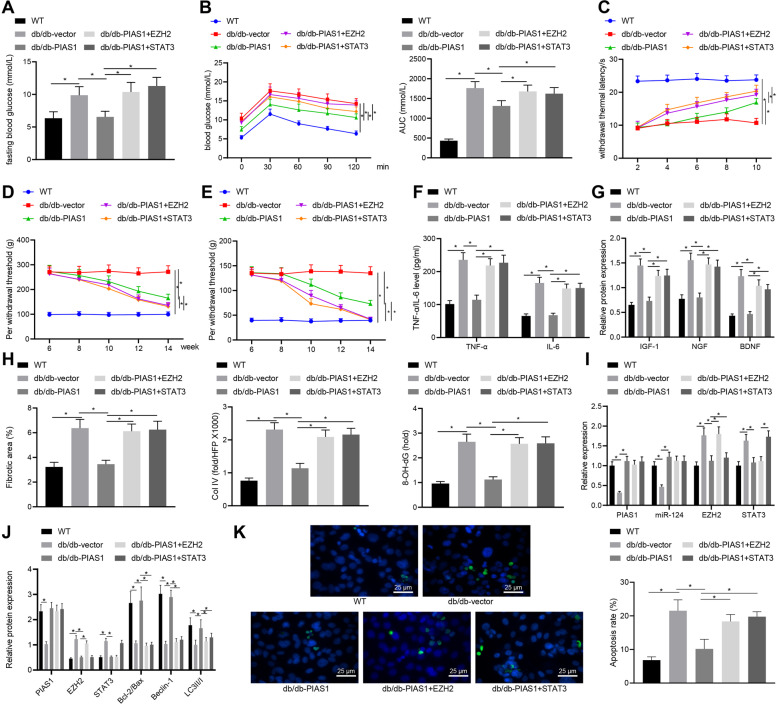
PIAS1 alleviates diabetic neuropathy in vivo. **A** Fasting blood glucose of mice with different treatment as detected by OGTT. **B** Glucose tolerance of mice with different treatment. **C** Thermal pain area of mice with different treatment. **D** Mechanical hyperalgesia of mice with different treatment as detected by Randall-Selitto paw pressure test. **E** Abnormal mechanical tactile pain of mice with different treatment. **F** The expression of pro-inflammatory cytokines TNF-α and IL-6 as measured by ELISA. **G** The expression of IGF-1, NGF, BDNF, which are important pathogenic factors for DPN, as determined by western blot assay. **H** Masson’s trichrome staining for nerve fibrosis of mice with different treatment. **I** The mRNA expression of miR-124, PIAS1, EZH2, and STAT3 in sciatic nerve tissue of mice with different treatment as determined by RT-qPCR. **J** The protein expression of PIAS1, EZH2, STAT3, Bcl-2, Bax, Beclin-1, and LC3II/I as determined by western blot assay. *n* = 6. **K** The apoptosis of cells evaluated by TUNEL staining. *p < 0.05. Data between two groups were compared by independent sample *t* test, and those among multiples by one-way ANOVA, followed by Tukey’s post hoc tests. The experiment was conducted in triplicates.

In order to further explore the function of PIAS1 in diabetic neuropathy, adenovirus stably overexpressing PIAS1, EZH2, and STAT3 was infected into db/db mice with spontaneous type 2 diabetes mellitus. The fasting glucose tolerance of mice with different treatment was measured and OGTT was performed to confirm the successful modeling (Fig. 6A). The results showed that PIAS1 overexpression could effectively reduce blood glucose, relieve thermal hyperalgesia, increase mechanical hyperalgesia and opioid-induced hyperalgesia, while all these changes were reversed upon overexpression of PIAS1 and EZH2 or overexpression of PIAS1 and STAT3 (Fig. 6B–E). The results of ELISA and western blot assay revealed that PIAS1 overexpression inhibited the expression of pro-inflammatory cytokines including tumor necrosis factor-α (TNF-α) and interleukin-6 (IL-6), as well as the pathogenic factors for diabetic peripheral polyneuropathy including insulin-like growth factor 1 (IGF-1), nerve growth factor (NGF) and brain-derived neurotrophic factor (BDNF), which was reversed by overexpressed PIAS1 and EZH2 or overexpressed PIAS1 and STAT3 (Fig. 6F, G). Masson’s trichrome staining showed that PIAS1 effectively inhibited the expression of Col IV in sciatic nerves and the continuous increase of fibrosis area. Relative to PIAS1 overexpression, overexpressed PIAS1 and EZH2 or overexpressed PIAS1 and STAT3 caused significantly increased fibrosis (Fig. 6H). The results of RT-qPCR and western blot assay showed that after overexpression of PIAS1, the expression of PIAS1, Bcl-2/Bax, Beclin-1, LC3II/I, and miR-124 was significantly increased, while that of EZH2 and STAT3 was markedly decreased. Compared with PIAS1 overexpression, overexpressed PIAS1 and EZH2 or overexpressed PIAS1 and STAT3 increased the expression of EZH2 and STAT3 while diminishing that of Bcl-2/Bax, Beclin-1, LC3II/I (Fig. 6I, J). Results of TUNEL staining further demonstrated that mice of the db/db-Vector group presented with increased cell apoptosis relative to those in the WT group, and this increase was then negated by PIAS1 overexpression. Whereas, additional restoration of STAT3 or EZH2 could reverse the suppressing effect of PIAS1 overexpression alone on cell apoptosis in db/db- mice (Fig. 6K). Collectively, PIAS1 can improve diabetic neuropathy by regulating the miR-124-EZH2/STAT3 pathway.

## Discussion

Half of patients with diabetes mellitus are suffering from DPN [22]. In the current study, we explored the regulatory mechanism of PIAS1 in DPN and found that PIAS1 inhibits the development of DPN by regulating the PPAR-γ-miR-124-EZH2/STAT3 axis.

Initially, we discovered downregulation of PIAS1 in DPN and demonstrated that PIAS1 enhanced the stability of PPAR-γ protein through SUMOlation, which contributed to an increase in the expression of miR-124. In relation to our finding, several previous studies have highlighted the role of PIAS1 in diabetes mellitus or neurological diseases. Notably, it has been revealed that the critical role of PIAS1 exerted important functions in controlling insulin sensitivity and thus might be potential for treatment of type 2 diabetes mellitus [11]. Moreover, PIAS1 could exert neuroprotection in hippocampus of mice with Alzheimer’s disease through SUMOylation of HDAC1 [23]. As previously reported by Xie et al., PIAS1 contributed to enhanced SUMO modification of PPAR-γ, which could exert protection against myocardial ischemia-reperfusion injury [12]. Lan et al. discovered the SUMOylation of PPARγ partly by PIAS1 in human umbilical vascular endothelial cells [24]. To our knowledge, PPARs play an important role in regulation of Schwann cells. PPAR agonists were found to prevent glucose-induced metabolic memory in Schwann cells [25]. In addition, PPAR-γ could modulate injury and regeneration of peripheral nerves by inducing differentiation of Schwann cells [14]. Moreover, the activated PPAR-α by fenofibrate could lead to improvement of neural and endothelial injury in db/db mice and human Schwann cells, which can inhibit the progression of DPN [26]. Overall, the above reports can support our finding that the inhibitory role of PIAS1 in DPN involves PPAR-γ SUMOlation and the resultantly upregulated miR-124.

Importantly, we found in this study that PPAR-γ promotes the expression of miR-124, which further downregulated EZH2 and STAT3 to promote the autophagy of Schwann cells. Intriguingly, the regulation of miR-124 by PPAR-γ has been reported. For instance, activated PPAR-γ could increase the expression of miR-124 in vitro and in vivo to reduce production of pro-inflammatory cytokines [15]. In addition, Liu et al. demonstrated that PPAR-γ could regulate miR-124 in pulmonary arterial smooth muscle cells [27]. Furthermore, an increasing number of studies have unfolded the alleviatory effects of miR-124 on diabetes mellitus-related diseases. miR-124a could lead to enhancement of therapeutic functions of bone marrow stromal cells on epithelial-to-mesenchymal transition and fibrosis related to diabetic nephropathy [28]. Furthermore, increased miR-124-3p in microglial exosomes was found to exert neuroprotective functions post traumatic brain injury by regulating neuronal autophagy mediated by FIP200 [29].

It is noteworthy that a large number of studies have unveiled the regulation of miR-124 on EZH2 and STAT3. As previously reported, miR-124-3p could target EZH2 to alleviate chronic sciatic nerve injury-induced neuropathic pain [30]. Moreover, it was found that miR-124 could exert a tumor inhibitory effect on multiple myeloma cell line by targeting EZH2 [31]. Consistently, a previous study revealed that miR-124 could target STAT3 to modulate microglial activation, thereby alleviating depressive-like behavior [32]. Our findings corroborate accumulating evidence illuminating the role of EZH2 and STAT3 in diabetes mellitus-related or neurological diseases. The repressed EZH2 was unfolded to aid in alleviating podocyte injury in diabetic nephropathy [33]. Besides, the recruitment of EZH2 was observed in the Egr2 promoter following sciatic nerve injury and plays an important role in the biology of peripheral nervous system [34]. Pregabalin and lacosamide ameliorate paclitaxel-induced peripheral neuropathy via inhibition of JAK/STAT3 signaling pathway and Notch-1 receptor [35]. Collectively, PIAS1-mediated PPAR-γ can upregulate miR-124 to inhibit EZH2 and STAT3, thereby regulating the metabolism of Schwann cells.

**Fig. 7:**
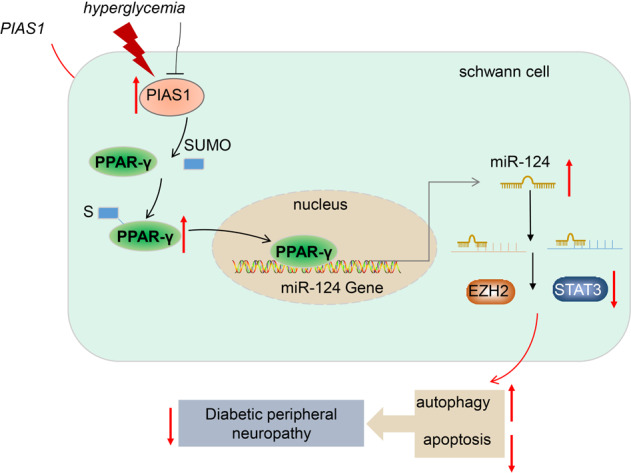
Molecular mechanism plot for PIAS1 in DPN. In DPN, PIAS1 expression is downregulated; apoptosis of Schwann cells is increased and autophagy is reduced. Overexpression of PIAS1 can promote the SUMOlation of PPAR-γ to stabilize its expression, thereby increasing the binding between PPAR-γ and miR-124 promoter to promote the expression of miR-124. The increased miR-124 inhibits the expression of EZH2/STAT3, thereby inhibiting apoptosis and promoting the autophagy of Schwann cells, which suppresses DPN.

Based on the results obtained in the current study, we conclude that PIAS1 stabilizes PPAR-γ expression by promoting its SUMOlation, which increases the binding of PPAR-γ with miR-124 promoter and thus upregulates miR-124, thereby decreasing the expression of EZH2/STAT3. Importantly, the downregulated EZH2 and STAT3 further inhibit cell apoptosis and promote the autophagy of Schwann cells, thereby suppressing the development of DPN (Fig. 7). This finding may provide a novel direction for treatment of DPN but still needs further confirmation to explore the clinical feasibility.

## Materials/subjects and methods

### Ethical approval

The animal experiments were performed under the approval of the animal ethics committee of The First Affiliated Hospital of University of South China and in accordance with the guidelines for the care and use of laboratory animals issued by the National Institutes of Health.

### Cell culture and transfection

A human embryonic kidney cell line (HEK293T) and human Schwann cells (SC4) were purchased from Beike Biotechnology (Shenzhen, China). HEK293T cells were cultured in Dulbecco’s modified Eagles medium (DMEM) (Gibco, Carlsbad, CA, USA) containing 10% fetal bovine serum (FBS; Gibco), and SC4 cells were cultured in SC medium (ScienCell Research Laboratories, San Diego, CA, USA), both in a cell incubator (Thermo Fisher Scientific, Rockford, IL, USA) at 37 °C with 5% CO_2_ and saturated humidity. In order to prevent bacterial contamination, penicillin/streptomycin antibiotics were added to the culture medium. The final concentration of penicillin was 100 U/mL, and that of streptomycin was 100 μg/mL.

SC4 cells were exposed to high glucose (40 mmol/L D-glucose) to construct an in vitro cell model as preciously described [36].

According to the requirements of Lipofectamine 2000 (Invitrogen, Carlsbad, CA, USA) transfection instructions, the cells were, respectively, transfected with plasmids overexpressing PIAS1/PPAR-γ/miR-124, or plasmids shuttling siRNA targeting PIAS1/PPAR-γ, or the combination of miR-124 overexpression plasmids and PPAR-γ-silencing/EZH2-overexpressing/STAT3-overexpressing plasmids. After transfection for 6 h, the medium was renewed for further culture. siRNAs or miRs were synthesized by Ribobio (Guangzhou, China), and the expression plasmids were purchased from GenePharma (Shanghai, China); the plasmid concentration was 50 ng/mL.

### Reverse transcription-quantitative polymerase chain reaction (RT-qPCR)

Trizol reagent (16096020, Thermo Fisher Scientific) was used to extract total RNA, For mRNA detection, cDNA was obtained using a reverse transcription kit (RR047A, Takara, Tokyo, Japan); for miRNA detection, a PolyA-Tailed Reverse Transcription Kit (B532451, Sangon Biotech. Shanghai, China) was utilized to synthesize cDNA. The synthesized cDNA was determined by RT-qPCR using a SYBR® Premix EX TAQTM II Kit (Drr081, Takara) in the ABI PRISM 7500 RT-PCR system (Applied Biosystems, Carlsbad, CA, USA). Using β-actin or U6 as internal reference, the relative expression of target genes was analyzed using the 2^−ΔΔCT^ method. The specific sequences are shown in Supplementary Table 1.

### Western blot assay

Cells were collected and washed with twice phosphate-buffered saline (PBS). Next, radioimmunoprecipitation assay (RIPA) buffer (Thermo Fisher Scientific) and protease inhibitor (Sigma-Aldrich Chemical Company, St Louis, MO, USA) were added to the cells (1 mL per 1 × 10^7^ cells), and the protein concentration was determined by BCA protein quantitative kits (Thermo Fisher Scientific). Subsequently, 20–60 μg protein samples were separated by 8–15% sodium dodecyl sulfate polyacrylamide gel electrophoresis. The separated proteins were transferred to a polyvinylidene fluoride membrane (Pall Life Sciences, Ann Arbor, USA), and blocked with 5% bovine serum albumin at room temperature for 2 h. The proteins were incubated overnight at 4 °C for 1 h at room temperature, with diluted primary antibodies against Flag (ab125243), hemagglutinin (HA) (ab9110), PIAS1 (ab109388), PPAR-γ (ab59256), EZH2 (ab186006), STAT3 (ab68153), phosphorylated (p)-STAT3 (ab32143), Bcl-2 (ab32124), Bax (ab32503), Beclin-1 (11306–1-AP), LC3 (ab192890), β-actin (ab8226), NGF (ab52918), BDNF (ab108319), IGF-1 (ab133542). All the antibodies were purchased from Abcam Inc. (Cambridge, MA, USA), except for that against Beclin-1 (Proteintech Group Inc., Chicago, IL, China). The following day, the proteins were incubated with corresponding fluorescence-labeled secondary antibodies (DyLight 800 Conjugated anti-Rabbit immunoglobulin G (IgG), EarthOx, Millbrae, CA, USA; DyLight 800 Conjugated anti-Mouse IgG, EarthOx). Odyssey infrared fluorescence scanning imaging system was used for the detection. Image J software was used to quantify the gray level of each band in western blot assay images, and β-actin was used as internal reference.

### Chromatin immunoprecipitation (ChIP)

ChIP kits (Millipore, Bedford, MA, USA) was used to collect cells. When the confluency of cells reached 70–80%, 1% formaldehyde was used to fix the cells at room temperature for 10 m to cross-link intracellular DNA and protein. After cross-linking, they were randomly broken by ultrasonic treatment, followed by centrifugation at 4 °C and 13000 rpm. The supernatant was placed into three tubes, which were respectively added with positive control antibody RNA polymerase II, NC antibody human IgG, and anti-PPAR-γ, followed by overnight incubation at 4 °C. The endogenous DNA protein complex was precipitated by Protein Agarose/Sepharose, and the supernatant was removed after a short time of centrifugation. Following washing the nonspecific complex, cross-linking was carried out for a whole night at 65 °C. The DNA fragments were extracted and purified by phenol/chloroform, and the expression of miR-124 promoter was determined by RT-qPCR.

### Co-immunocoprecipitation (Co-IP)

The cultured cells were collected and treated with 1 mL IP lysis buffer containing protease inhibitor on ice for 15 m. Centrifugation was performed at 12,000 rpm for 10 m, followed by transfer of the collected supernatant into a new centrifuge tube. The lysate was mixed with specific antibodies against Flag, HA or IgG, and 25 μL protein A Sepharose, followed by overnight incubation at 4 °C. The beads were rinsed three times with IP lysis buffer. The same volume of 2 × sodium dodecyl sulfate buffer solution was added to the beads, followed by mixing and heating at 95 °C for 10 m. After centrifugation, the supernatant was collected and the expression of PIAS1 and PPAR-γ was analyzed by western blot assay.

### Dual-luciferase reporter gene assay

The predicted binding site fragments (miR-124 with EZH2 and STAT3) and mutation fragments were inserted into the dual-luciferase reporter vector as the reporter plasmids, which were labeled as wild type (WT) and mutant type (MUT), respectively; Renilla fluorescence served as internal reference. Luciferase reporter vectors were co-transfected with miR-124 mimic or mimic NC respectively. After transfection for 6 h, 400 μL of complete culture medium containing 10% FBS was added to the cells for further culture with 5% CO_2_ at 37 °C for 24 h. The cells were collected according to the protocol of dual-luciferase detection kits (E1910, Promega Corporation, Madison, WI, USA). Next, 200 μL of 1× PLB was added into the cell culture wells to lyse the cells, and the cell lysate was transferred to a white board (20 μL/well) for detection. The luciferase activity was measured using a Tecan M1000 Microplate reader (Tecan, Mnnedorf, Switzerland).

### Flow cytometry

The cells were washed twice with PBS, detached with trypsin without ethylene diamine tetraacetic acid, and centrifuged at 2000 rpm (~400 × *g*) for 5 min. Next, 500 μL of binding buffer suspension were added to resuspend the cells. A total of 5 μL Annexin V-fluorescein isothiocyanate (FITC) was added into the suspension, followed by addition of 5 μL propidium iodide (PI). The mixture was reacted in room temperature for 15 m in darkness. A flow cytometry (BD Biosciences, Franklin Lakes, NJ, USA) was used to measure the green fluorescence of Annexin V-FITC through FITC channel and red fluorescence of PI through PI channel.

### Enzyme-linked immunosorbent assay (ELISA)

ELISA kits (EK0411, EK0527, Boster, Wuhan, China) of TNF-α and IL-6 were used for the experiment, and ELISA was conducted following the instructions of the kits. The optical density at 450 nm wavelength was measured using a Microplate reader. Based on the optical density of the standards, standard curves were drawn to calculate the sample concentration.

### Animal experiment

The db/db mice, ob/ob mice, and C57BL/6 WT mice purchased from Vital river (Beijing, China) were raised in a specific-pathogen-free animal room. The expression of PIAS1 in sciatic nerve tissues of in db/db mice, ob/ob mice and C57BL/6 WT mice was determined. Twenty-four 8-week-old male db/db mice were randomly assigned into four groups with 6 mice in each group. Six male C57BL/6 WT mice aged 8 weeks old were selected as NCs (marked as WT). After 4 weeks of feeding, the first group was injected with adenovirus carrying blank control (labeled as db/db), the second with adenovirus overexpressing PIAS1 (labeled as PIAS1), the third with adenovirus overexpressing PIAS1 and EZH2, and the fourth with adenovirus overexpressing PIAS1 and STAT3. The experiment lasted for 4–8 weeks.

Measurement of fasting blood glucose: the mice were fasted for 6 h, with free access to water, after which the glucose concentration in tail vein blood was measured using a blood glucose meter (Roche Diagnostics GmbH, Mannheim, Germany).

OGTT: the mice were fasted for 6 h, with free access to water, after which they were intragastrically administered with 1 g/kg glucose. The glucose concentration in tail vein blood of mice was measured using a blood glucose meter at 0, 30, 60, 90, 120 m.

Measurement of thermal pain area: an intelligent hot plate instrument was used to for the measurement, and the interval between each measurement was at least 15 m. Measurement of mechanical hyperalgesia (Randall-Selitto paw pressure test): before the experiment, mice were allowed to stand on the Ugo Basile dynamic plantar anesthesia apparatus for 20 m to adapt to the surrounding environment. The cut-off time was set at 30 s, when a constant force of 20 g/s was applied to the injected claw, and the reaction time of injurious behavior (withdrawal of injection claw) was recorded every hour for 5 h.

Measurement of tactile mechanical pain (Von Frey hair test): mechanical withdrawal threshold was measured using 0.02–0.2 g von Frey filaments to evaluate mechanical sensitivity. The mice were placed on the wire mesh floor alone, covered with an opaque chamber, and allowed to adapt to the new environment for at least 30 m before the test. Subsequently, von Frey filaments were applied to the plantar surface of the hind paw. Finally, 50% paw withdrawal threshold was determined by up-down method.

### Histopathological analysis of sciatic nerve

The sciatic nerves of mice were harvested and stored in 10% formalin for 24 h. The samples were dehydrated and immersed in xylene for 1 h (three times), and then in ethanol (70, 90, and 100%) for 2 h. The tissue specimens were cut into 3- to 5-μm sections. Masson’s trichromatic staining was used to examine nerve fibrosis, reflected by collagen type IV (Col IV; Biosign international, Saco, ME, USA) and DNA damage marker 8-hydroxy-deoxyguanosine (8-OH-dG; Jalca, Fukuroi, Japan).

### TUNEL staining

The cell apoptosis was detected using a TUNEL staining kit (11684817910, Roche Diagnostics GmbH). After dehydration, the cells were incubated with the TUNEL reaction mixture for 1 h at 37 °C, followed by PBS washing and removal of residual liquid. The number of apoptotic cells was counted using a fluorescence microscopy. Specifically, the cells were separately incubated with converter-POD (horseradish peroxidase-labeled fluorescein antibody) and DAB substrate, and the TUNEL-positive cells were then quantified, with two independent pathologists who were blinded with the groups to count the positive cells in five fields of view.

### Statistical analysis

All the data in this study were analyzed with the use of the SPSS 21.0 statistical software (SPSS, IBM, Armonk, NY, USA). The measurement data were expressed by mean ± standard deviation. Data between two groups were compared by independent sample *t* test, and those among multiples by one-way analysis of variance (ANOVA), followed by Tukey’s post hoc tests. *p* < 0.05 was indicative of statistically significant difference.

## Supplementary information

Supplementary Table 1

author-contribution-form

## Data Availability

The data that supports the findings of this study are available in the manuscript and supplementary materials.
