# Supplementary material for: PIAS1 alleviates diabetic peripheral neuropathy through SUMOlation of PPAR-γ and miR-124-induced downregulation of EZH2/STAT3
Source: Cell Death Discov. 2021 Dec 2;7:372. doi: 10.1038/s41420-021-00765-w (PMC8639830; doi:10.1038/s41420-021-00765-w)
Supplement: Supplementary file 1 — Supplementary Table 1 [file 41420_2021_765_MOESM1_ESM.docx]

**Supplementary Table 1** Primer sequences for RT-qPCR

| Gene | Sequence |
| --- | --- |
| EZH2 | F: 5'-GGACTCAGAAGGCAGTGGAG-3' |
|  | R: 5'-CTTGAGCTGTCTCAGTCGCA-3' |
| STAT3 | F: 5'-TCTGCCGGAGAAACAGTTGG-3' |
|  | R: 5'-AGGTACCGTGTGTCAAGCTG-3' |
| U6 | F: Universal primer |
|  | R: Universal primer |
| miR-124 | F: 5'-GGTATCCACTGTAGGCCTATATG-3' |
|  | R: Universal primer |
| β-actin | F: 5-CATGTACGTTGCTATCCAGGC-3' |
|  | R: 5-CTCCTTAATGTCACGCACGAT-3' |

**Note:** EZH2, enhancer of zeste homolog 2; F, forward; R, reverse; STAT3, signal transducer and activator of transcription 3; miR-124, microRNA-124; RT-qPCR, reverse transcription-quantitative polymerase chain reaction
