# Supplementary material for: PIAS1 alleviates diabetic peripheral neuropathy through SUMOlation of PPAR-γ and miR-124-induced downregulation of EZH2/STAT3
Source: Cell Death Discov. 2021 Dec 2;7:372. doi: 10.1038/s41420-021-00765-w (PMC8639830; doi:10.1038/s41420-021-00765-w)
Supplement: Supplementary file 2 — author-contribution-form [file 41420_2021_765_MOESM2_ESM.pdf]

DECLARATION OF CONTRIBUTIONS TO ARTICLE

ADMC

Manuscript Number: CDDISCOVERY-21-1616

Journal Name: *Cell Death Discovery* (the 'Journal')

Proposed Title of the Contribution:

PIAS1 alleviates diabetic peripheral neuropathy through SUMOlation of PPAR-γ and miR-124-induced downregulation of EZH2/STAT3 (the 'Contribution)

Author(s):

Zixin Hou, Ji Chen, Huan Yang, Xiaoling Hu, Fengrui Yang (the 'Authors')

For all *CDDis*covery articles, each person named as an author in the published version must be able to show he or she has contributed substantially to the article.

Authorship credit should be based on 1) substantial contributions to conception and design, acquisition of data, or analysis and interpretation of data; 2) drafting the article or revising it critically for important intellectual content; and 3) final approval of the version to be published. Authors should meet conditions 1, 2 and 3.

Any person who cannot be shown to have made a substantial contribution to the article cannot be listed as an author in the final version. The name of any person who is deemed to have made a minor contribution can, however, appear in the Acknowledgments section of the article.

Please complete the table below to indicate the contributions of all named authors to the manuscript.

Author Full Name:	Specification of Contribution to the Manuscript:
Zixin Hou	conceived and designed research
Ji Chen	performed experiments and prepared figures
Huan Yang	interpreted results of experiments and analyzed data
Xiaoling Hu	drafted paper
Fengrui Yang	edited and revised manuscript

Please complete the table below to indicate the contributions of all named authors to the figures.

Figure 1:

Ji Chen

Figure 2:

Ji Chen

Figure 3:

Ji Chen

Figure 4:

Ji Chen

Figure 5:

Ji Chen

Figure 6:

Ji Chen

Signed for and on behalf of the Author(s):	Print Name:	Date:
Fengrui Yang	Fengrui Yang	2021/7/5
